# Method of Mounting Gum Teeth on Rubber and Protecting the Joints

**Published:** 1869-10

**Authors:** Jas. Gordon

**Affiliations:** St. Thomas


### ARTICLE IV.

### *Method of Mounting Gum Teeth on Rubber and Protecting the Joints.*

By JAS. GORDON of St. Thomas

When I first began to practice the insertion of gum teeth upon rubber base, I experienced great difficulty in preventing the rubber from getting between the joints; I tried all devices I have seen recommended in different dental communications.

In some cases I succeeded by filling behind the joints with tin foil, but even this I could not always rely upon. At first I thought it was from want of skill on my part, but, after the lapse of time, when I had opportunities to examine a great many pieces, I became persuaded that the difficulty did not rest with me alone. I have seen the rubber between the joints of many finely executed dentures, marring the full appearance of the teeth and which, I am certain, must have been of great annoyance to the operator.

There is nothing more pleasing in rubber work, than to see a finished piece with the seams nicely brought together and perfectly clean. It is true that when block teeth are used, in a complete denture, the seams are few and one would not be much observed; but to our great regret after the piece is completed, we behold the sightly appearance of *dark seams* in connection with the beautiful mineral gum, which is a reproach to dental mechanism.

What I found most effectual and without a single case of failure, is to grind the teeth as for plate work, having no V-shapes behind the joint as recommended by a great number of the profession, and in the process of packing to put behind each seam a strip of English pink rubber, such as is manufactured by Messrs. C. Ash & Sons, London. This rubber it is well known, owing to the percentage of earthy matters it contains, does not flow, if I may be allowed this term, into irregularities like the darker rubbers. This quality renders it the very thing suitable to protect the joints from being filled with rubber.

If I may be allowed to trespass on your time a little longer, I would beg to mention that it is essential to use just enough rubber that the halves of the flask may be brought together, without causing any undue lateral pressure; as it is experienced and generally known if too much pressure be used the teeth will in most casas spread apart and destroy the articulation of such a substitute. The most expiditious method I have adopted in the process of packing, is to ascertain from the base plate by any of the known methods, the quantity of rubber required for the case, and after packing as usual, interpose a piece of muslin between the mould and the rubber. This will facilitate the opening of the flask again, after it has been screwed down, to see if the case is properly packed. If there be not enough rubber add some, but if you observe that there is too much, trim away the surplus with a pair of scissors, remove the muslin, screw down as usual and the case is ready to vulcanize. This process can also be executed without ascertaining the quantity of rubber required; but the first is preferable.

In difficult cases of moulds with undercuts in which the manipulator apprehends the opening of the flask, the following device will be all that is needed:—In placing the mould in the flask fix it so that the undercuts remain in the lesser section, in such a manner that when the flask is opened, the undercuts in the mould can be packed apart from the upper section.

Next proceed to pack the undercuts, after which cover the exposed surface of the rubber with a piece of muslin or, which is better, a piece of tin foil, and when the upper section is also packed, interpose a piece of muslin again and proceed as before, of course interposing two pieces of muslin or muslin and tin to prevent the adhesion of the upper rubber with that of the undercuts.

By this method single gum teeth can in all cases be used with perfect success.

